# The dynamics and determinants of specific systemic and mucosal antibody responses to SARS-CoV-2 in three adult cohorts in the Ecuadorian Andes: a study protocol

**DOI:** 10.12688/f1000research.126577.1

**Published:** 2022-11-28

**Authors:** Jose E. Leon-Rojas, Tatiana Veloz, Jair Teran, Monica Perez, Fernanda Arias-Erazo, Lizet Villacis, Jorge Velez, Ricardo Recalde, Patricia Jiménez, Miguel Martin, Irina Chis Ster, Philip Cooper, Natalia Romero

**Affiliations:** 1 Departamento de Pediatría, Obstetricia y Ginecología y Medicina Preventiva, Universitat Autonoma de Barcelona (UAB), Barcelona, Spain; 2 Red Grups de Recerca d’Amèrica i Àfrica Llatines (GRAAL),, Quito, Ecuador; 3 Medical School, Universidad Internacional del Ecuador, Quito, Ecuador; 4 Departamento de Ciencias de la Vida y de la Agricultura, Universidad de las Fuerzas Armadas ESPE, Sangolquí, Ecuador; 5 Medical School, Universidad Técnica de Ambato, Ambato, Ecuador; 6 Hospital de Especialidades Eugenio Espejo, Quito, Ecuador; 7 Grupo de Investigación en Sanidad Animal y Humana (GISAH), Quito, Ecuador; 8 St George's University of London, London, UK

**Keywords:** COVID-19, SARS-CoV-2, Seroprevalence, Antibody levels, risk factors, Ecuador

## Abstract

**Introduction:** There are limited longitudinal data on the systemic and mucosal antibody responses to SARS-CoV-2 from Latin America, a region severely affected by COVID-19, and where vaccine strategies have been implemented during the evolving pandemic.

**Objective:** To evaluate determinants of seroprevalence and changes in levels of anti-SARS-CoV-2 antibodies longitudinally in adults with different levels of exposure to SARS-CoV-2 (defined *a priori* as low, medium, and high based on presumed occupational risk), in two Andean cities in Ecuador.

**Methods:** Longitudinal cohort study of 1,000 adults aged 18 years and older with questionnaire data and sample collection done at 0, 3, 6, and 12 months during the period 2020-2023. Observations collected included WHO-ISARIC questionnaire and peripheral blood and saliva samples for measurement of IgG and IgA antibodies, respectively. Planned analyses are tailored to the longitudinal nature of the outcomes defined by participants’ antibody levels and aim at estimating their average trends with time since infection in each of the occupational groups, adjusted for demographics and calendar-time levels of SARS-CoV-2 infection in the general population. The latter reflect the impact of the national control measures such as vaccinations and movement restrictions.

**Importance:** Understanding the duration and the dynamics of waning immunity to SARS-CoV-2, in the context of exposures to emerging virus variants and immunization, will inform the implementation of targeted public health strategies in the Latin American region.

**Ethics and Dissemination:** This study will observe the bioethical principles of the Declaration of Helsinki. Informed written consent will be obtained. Samples from participants will be stored for up to three years after which they will be destroyed. The study protocol was approved by the Ecuadorian Ministry of Public Health Ethics Committee for COVID-19 Research. Antibody results will be provided to participants and participating institutions and to the national health authorities.

## Introduction

The pandemic caused by the new coronavirus, SARS-CoV-2, the etiological agent of COVID-19, has caused 612 million confirmed cases and approximately 6.5 million deaths worldwide since December 2019 when the virus first emerged ( Johns Hopkins Coronavirus Resource Center). In Ecuador, the first confirmed case of COVID-19 was reported on 29 ^th^ February 2020 and as of 19 ^th^ September 2022, one million confirmed cases have been reported with 35,885 deaths. The highest burden of infection is among those aged 20 to 55 years age group, while death rates are greatest among those aged over 65 years ( Daily Report of the Ecuadorian Ministry of Public Health).

The presence of specific IgG antibodies to SARS-COV-2 indicates past exposure to SARS-COV-2 and vaccination, given that most serological assays using the spike protein cannot distinguish between these two forms of exposure. Additionally, since the virus invades the body through the mucosal surfaces, the role of mucosal immune responses measured by specific IgA, has been neglected compared to the measurement of systemic IgG responses. ^1^ Timely seroprevalence studies in the population are useful in monitoring rates of exposure to natural infections (prior to vaccination programs), and vaccine coverage and immunogenicity following the implementation of vaccination programs. ^2^ Measuring the waning immunity across different groups in the population will add to our understanding of the epidemiology of the infection and will help identify population groups with increased infection susceptibility. ^1^^–^^3^

Numerous seroprevalence studies have been conducted in the Latin American region, both population-based studies and studies focusing on specific populations (healthcare (HCW) or first-line workers, prison workers, indigenous populations, elderly populations, etc). ^4^^–^^15^ The reported seroprevalence is highly heterogenous between studies (10,4% – 68,1 %), varying by study population, characteristics of the COVID-19 epidemic at the time of sampling, and differences in sociodemographic factors. ^4^^–^^15^ Factors associated with increasing risk of seropositivity include ethnicity (indigenous or Afro-descent), presence of COVID-19 symptoms, past history of COVID-19 or close contacts, lower educational level, and working night shifts (for HCW) or in a prison. ^4^^–^^15^

Among these studies, a cross-sectional study in 2020 of 3,124 children aged 5 to 17 years in 10 Colombian cities reported highly variable seroprevalence ranging from 25.0% in Medellin to 63.9% in Guapi, the latter a coastal city with a high proportion of Afro-Colombians with the poorest living conditions. ^10^ Further, a cross-sectional study in 2021 from Brazil of six indigenous groups in the Amazon region showed a high average seroprevalence for anti-SARS-CoV-2 IgG (68.1%). ^11^ Finally, a systematic review analysing 15 seroprevalence studies from Latin America and the Caribbean reported a highly variable seroprevalence between indigenous populations ranging 4.2% to 81.7%. ^13^

There are nine published studies estimating seroprevalence rates for IgM/IgG antibodies to SARS-CoV-2 from different regions of Ecuador ( Table 1) covering the period from April 2020 to April 2021 with seroprevalence estimates ranging 7.7% to 64%. ^16^^–^^24^ Six of these studies are derived from samples nested within the same observational adult cohort from Atahualpa, a coastal community in Santa Elena Province and it is uncertain the degree of overlap between samples. ^16^^–^^21^ Two of four longitudinal studies using the Atahualpa cohort with follow-up of 1 ^16^ and 12 ^18^ months, estimated seroincidence (i.e., incidence rate ratio for seroconversion) of 7.4 ^16^ to 9.9 ^18^ per 100 person years, respectively.

**Table 1.** Summary of seroprevalence estimates for IgM/IgG antibodies to SARS-CoV-2 from Ecuador, ordered by sampling date.

Author (Year)	Location	Design	Date of Sampling	Sample size	Age (mean yrs)	Test	Seroprevalence
Del Brutto (2021) ^21^	Atahualpa, Santa Elena Province, coastal rural	Longitudinal	April 2020 - May 2021	277	70.6	IgM/IgG Rapid Test	63.2% (95% CI 57.4-68.6)
Del Brutto (2020) ^17^	Atahualpa, Santa Elena Province, coastal rural	Cross-Sectional	May 2020	673	59.2	IgM/IgG Rapid Test	45%
Del Brutto (2020) ^20^	Atahualpa, Santa Elena Province, coastal rural	Cross-Sectional	May 2020	319	70.5	IgM/IgG Rapid Test	44%
Del Brutto (2020) ^16^	Atahualpa, Santa Elena Province, coastal rural	Longitudinal	May-June 2020	362	59.9	IgM/IgG Rapid Test	7.7% IRR 7.4/100 person months (95% CI 4.7-10.2)
Del Brutto (2021) ^19^	Atahualpa, Santa Elena Province, coastal rural	Longitudinal	May 2020 – April2021	519	61.8	IgM/IgG Rapid Test	63%
Del Brutto (2021) ^18^	Atahualpa, Santa Elena Province, coastal rural	Longitudinal	May 2020 - April 2021	673	59.2	IgM/IgG Rapid Test	64% IRR 9.9/100 person months (95% CI 8.9-10.8)
Acurio-Páez (2021) ^22^	Cuenca, Azuay Province, Andes, rural/urban	Cross-Sectional	Aug -Nov 2020	2,457	39.0	IgM/IgG Rapid Test	13.2% (95% CI 12.0-14.6%)
Zambrano (2021) ^23^	Babahoyo, Guayas Province, coastal urban	Cross-Sectional	Sept 2020 - Jan 2021	100 pregnant women	24.8	IgM/IgG ELISA	32%
Vallejo-Janeta (2022) ^24^	5 coastal districts in Esmeraldas, Province	Cross-Sectional	Oct 2020	1250	45.3	IgM/IgG Rapid Test	11.7% (95% CI: 10.0-13.6);

IRR - incidence rate ratio for seroconversion; yrs - years; 95% CI - 95% confidence intervals.

**Figure 1.**
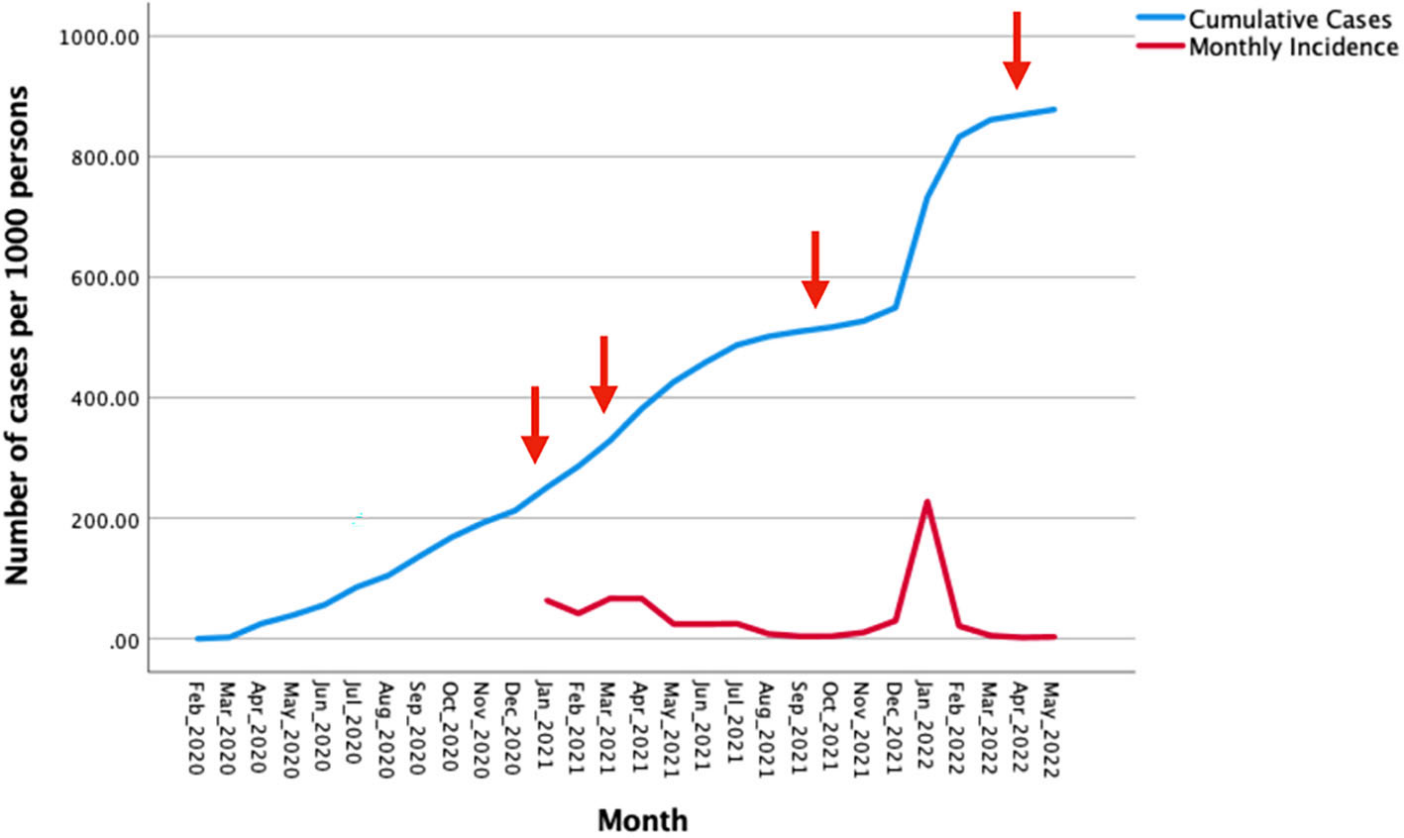
Confirmed cumulative SARS-COV-2 infections and monthly incidence per 1000 population over the period February 2020 to May 2022. Legend: Arrows indicate the initiation of different phases in the national vaccination programme. Data from: https://www.salud.gob.ec/coronavirus-covid19-ecuador/.

In most regions, vaccination has been a major component of national mitigation strategies for COVID-19 resulting in marked reductions in hospitalizations and deaths worldwide. ^25^ In Ecuador, vaccination was initiated on 21 ^st^ January 2021 as part of the national vaccination strategy “Plan Vacunarse”, consisting of four phases: Phase 0, a pilot phase, started in January 2021 and targeted first line workers and very high-risk individuals; Phase 1 started in March 2021 and included individuals with risk of severe disease such as the elderly; Phase 2 started in June 2021 and included the adult populations living in provinces with a high incidence of COVID-19; and Phase 3 started in September 2021 including adult populations in the other provinces. ^26^ Booster doses (third and fourth doses) started in October 2021 and April 2022, respectively ( Vaccination Report Ministry of Public Health). As of May 2022, 83% of the population had received a complete 2-dose vaccination schedule (with one of CoronaVac, Pfizer-BioNTech, AstraZeneca, and Cansino), and 35.6 % has received at least one booster shot ( Vaccination Report Ministry of Public Health). Figure 1 shows confirmed cumulative SARS-COV-2 infections and monthly incidence per 1000 population over the period February 2020 through May 2022.

Here, we present the protocol for a longitudinal study of seroprevalence and changes in systemic (plasma IgG) and mucosal (salivary IgA) antibodies being done in three adult cohorts of different infection risk justified by presumed different infection patterns, in two Andean cities. In addition, we will explore the effects of natural infections and vaccinations, as well as other relevant determinants (e.g. age, sex, and occupation), on seroprevalence and time trends since infection on antibody levels.

## Protocol

### General objective

To estimate changes in and determinants of seroprevalence and levels of anti-SARS-CoV-2 antibodies over 12 months of follow-up in adults aged 18 years and older, with varying levels of exposure (defined *a priori* as low, medium, and high based on presumed occupational risk) and associated factors, in two Ecuadorian Andean cities.

### Specific objectives

1. Identify risk factors (including cohort membership) associated with baseline seroprevalence and IgG antibody levels in the three cohorts (pre-vaccination)
2. Evaluate changes in seroprevalence and IgG antibody levels over at least 12 months of follow-up.
3. Identify effects of determinants on longitudinal changes in specific IgG antibody levels over at least 12 months of follow-up. Potential determinants will include cohort membership, sociodemographic factors, vaccination, number of vaccine doses, natural (past exposures) infections, and their timing.
4. Estimate the pattern of decline in anti-SARS-CoV-2 IgG antibodies among seropositives and impact of vaccinations and other relevant determinants on this decline.
5. To estimate the development and longevity of the mucosal immune response through the measurement of levels of anti-SARS-CoV-2 IgA in saliva and the impact of relevant determinants on changes in antibody levels.

### Methods

*Study design*

**Figure 2.**
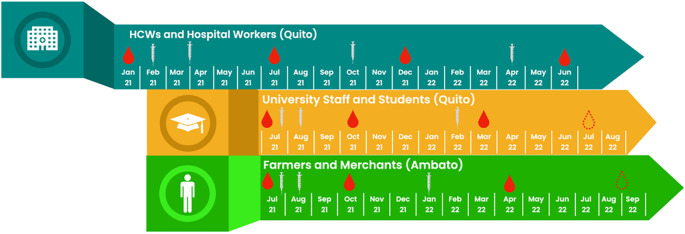
Recruitment and follow-up of 3 cohorts by calendar time. Legend: Syringes show introduction of COVID-19 vaccine doses, red drops show sample collections, and dotted red drops show timings of future sample collections.

Prospective longitudinal cohort ( Figure 2).

### Definition of the study population and eligibility criteria

Three cohorts from two Ecuadorian Andean cities, based on assumed risk of occupational exposure to SARS-CoV-2, are being recruited in two Andean cities, Quito, and Ambato. These three cohorts are presumed to differ in the probability of exposure to SARS-CoV-2. Eligibility criteria for entry into the 3 cohorts are provided in Table 2.

1. High Risk (HR): Defined as being of high exposure risk through an occupational setting of health care workers and support personnel working in the emergency service, ICUs and hospitalization areas at a major public hospital (Hospital Eugenio Espejo) in Quito, at altitude of 2,800 m in northern Andean region, with 2.9 million inhabitants.
2. Medium Risk (MR): Population whose occupation puts them at a considerable risk of contracting SARS-CoV-2 (merchants, farmers or those that do not self-isolate due to the nature of their work). The population selected was Huachi Grande parish in the city of Ambato, at altitude of 2,600 m, in central Andean region, with 500,000 inhabitants.
3. Low Risk (LR): Population with a presumed low risk of SARS-CoV-2 infection due to the characteristics of their occupation (staff and students at a private university, with the means to self-isolate) and living within the metropolitan district of Quito. This cohort is composed of teachers, administrative staff, and the student community of Universidad Internacional del Ecuador (UIDE), Quito.

**Table 2.** Eligibility criteria for three adult cohorts.

Characteristic	Cohort 1	Cohort 2	Cohort 3
Level of risk at recruitment/Setting/study population	High Risk: Health care workers in public hospital	Medium risk: merchants, farmers, and highly mobile workers	Low risk: staff and students at private university
Inclusion criteria	1. Adults aged 18 years or older 2. Healthcare workers 3. Hospital workers	1. Adults aged 18 years or older 2. Inhabitant of Huachi Grande	1. Adults aged 18 years or older
Exclusion criteria	1. Age <18 years 2. Pregnancy or breastfeeding 3. Religious beliefs that prevent participation 4. Unable to provide informed consent		

### Data and sample collection

*Questionnaires*

Data are being collected digitally by trained interviewers using a standardised tool developed specifically for the COVID-19 pandemic ( ISARIC-WHO Global COVID-19 Clinical Platform “Rapid core case report form (CRF)” questionnaire, subsections 1b, 1f, and 3a) at baseline (month 0) and every three months either via telephone or face-to-face interviews. This tool collects data on socio-demographics, history of signs and symptoms related to COVID-19, and previous diagnoses and treatments.

### Clinical samples

Saliva and blood are being collected at baseline and 3-monthly intervals (0, 3, 6, and 12 months). Blood samples are being collected into 5 ml Vacutainer tubes with EDTA as anticoagulant (Vacutainer, BD Biosciences), and plasma separated by centrifugation and stored in 0.5 mL aliquots at -20 °C. Saliva sample (0.5-1 mL) are being collected directly into 2 ml microtubes after asking the participant to think of food, centrifuged to remove residual food debris, and stored in 0.5 mL aliquots at -20 °C.

### Measurement of anti-SARS-CoV-2 IgG and IgA antibodies

Presence and levels of IgG antibodies directed against the Receptor Binding Domain (RBD) of the SARS-CoV-2 spike protein will be measured in stored plasma using an ELISA essay adapted from Guevara *et al.*, 2021. ^27^ Specific IgA antibodies against SARS-CoV-2 RBD will be measured in saliva samples using an ELISA-based assay modified from Costantini *et al.*, 2022. ^28^ Specific IgG and IgA quantitation will use standard curves of serum pools of positive samples calibrated against an WHO international standard (National Institute for Biological Standards and Control, Potters Bar, United Kingdom).

### Follow up

The initial sample was collected before vaccination in all cohorts; after baseline evaluation subsequent sampling was done as follows:

a. at 3 months after baseline if the participant had not been vaccinated.
b. at 30-45 days after receiving the two-dose vaccine schedule or boosters’ doses.

Follow-up was done on site (university, hospital, or parish administrative building) or through home visits at the sampling follow-up times (0, 3, 6, and 12 months depending on vaccine dose timings). Subjects were contacted also by phone at monthly intervals to collect data on new infections and vaccinations and to provide antibody results, as well as address any doubts or concerns about the study. All evaluations were done by a dedicated and trained study team.

### Statistical considerations

At the time of study planning, there were no reliable longitudinal data to estimate minimum sample size needed to understand the dynamics of waning immunity in different risk populations. Given the rapid dynamics of unfolding events, convenience sampling will be done in each of the three occupational risk cohorts with the aim of recruiting a total of 1,000 adults. Our data are exploratory in nature and the estimates will serve as input for planning future prospective comparative studies.

Cross sectional variables will be summarized according to their nature, i.e. means, standard deviations, medians, inter-quartiles, ranges and frequencies and proportions for categorical variables. Data summaries will be done for each risk group to understand the characteristics of each risk group.

Presence and levels of IgG/IgA antibodies will define binary and continuous longitudinal outcomes, respectively. Generalized estimation equations (GEEs) will be used to fit population averaged models under the most general assumptions of unstructured variance covariance matrix. It is likely that the distributions IgG/IgA antibody levels will be highly skewed and logarithmic transformation will be required to satisfy such models’ assumptions. The continuous longitudinal outcome will quantify patterns in trends in the monthly average waning immunity across groups and their characteristics – trends are expected to be nonlinear. The binary outcomes will evaluate the associations between presence/absence of antibodies and epidemiologically plausible independent variables through odds ratios. Mixed models will also be done and the estimates will be compared with those obtained by the GEEs fit. All analyses will be adjusted for levels of the infection in the general populations by calendar time, and by associated periods of interventions. Time-varying covariates are expected to be defined based on these later interventions. P-values less than 0.05 will indicate statistically significant (potentially adjusted) associations and 95% confidence intervals will assess the uncertainty of the estimates.

Multivariable models will be built for each risk group accounting for all potential explanatory variables and their interactions - particularly with time at follow-up. The most parsimonious model will be constructed based on the largest complete observations dataset using likelihood criteria such as quasi-likelihood value under the independence model criterion (QIC) for GEEs or likelihood-ratio type criteria for mixed models. GEE estimation is based on completely at random missing observations assumption whilst mixed models are based on missing at random assumption. The attrition patterns will be investigated, and sensitivity analyses to the estimates obtained on complete datasets using the two estimation techniques will be performed and discussed.

### Ethics and dissemination

The study protocol was approved by the Ecuadorian Ministry of Public Health Ethics Committee for COVID-19 Research (approval ID 020-2020- MSP-CGDES-2020-0172-O). The study results will be disseminated through presentations at conferences and to key stakeholder groups including policy makers, and through postgraduate theses and peer-review publications. All participants were required to accept participation by reading and signing an informed consent at the time of the first sampling.

## Discussion

We are following up three adult longitudinal cohorts with presumed distinct exposure risks to SARS-CoV-2 based on occupation, in two Andean cities of Ecuador, to study determinants of seroprevalence and changes in systemic and mucosal antibody levels against SARS-CoV-2.

Seroprevalence testing can be done using the detection of specific IgG and or IgM antibodies. IgM antibodies are detectable first from day 5-7 days post-infection with a peak at day 28 while IgG become detectable after 5-10 days and peak at 49 days. ^1^^,^^29^^,^^30^ In contrast, specific IgG antibodies following vaccination against SARS-CoV-2 are detectable from about a week following the first dose and peak at approximately 30 days after the second dose, remaining detectable for 3-6 months. ^31^^,^^32^ For example, a cross-sectional study in 56,261 Chilean individuals reported an IgG seroprevalence of 28.1% three weeks after the first dose and 77.4% four weeks after the second dose of the CoronaVac vaccine; and a prevalence of 79.4% three weeks after the first dose and 96.5% four weeks after the second dose of the BNT162b2 Pfizer vaccine. ^7^ In healthcare workers (HCW) receiving the BNT162b2 Pfizer vaccine, a study showed that median serum levels of antibodies reached a peak 1 month after the second dose and were 37% and 57% lower at 3 and 6 months, respectively. ^33^ Finally, a study, also in HCW, showed that antibodies remained high at three months after the third vaccination dose and that antibodies were quantitatively higher in those HCWs that had a history of COVID-19 infection and a third vaccine dose. ^34^

Our study aims to measure changes in antibody levels in the context of continuous (documented) exposures to natural infection and widespread vaccination with blood collections done as follows: 1) before the first vaccine dose; 2) at 30-60 days after the second dose; 3) 30-60 days after the third dose (when possible); and 4) 30-60 days after the fourth dose (where possible). These sampling times will allow us to measure effects of COVID-19 vaccination and booster doses with the three vaccines licensed for use in Ecuador (CoronaVac, Pfizer and AstraZeneca) on seroprevalence and antibody levels.

Other than vaccination or natural infection, additional factors have been reported to have significant effects on anti-SARS-Cov-2 antibodies. Front-line professionals such as doctors, nurses, hospital workers, police officers, among others, are more likely to acquire COVID-19 and therefore, would be expected to have higher seroprevalence rates than the general population. ^35^^–^^37^ A study involving 569 Canadian HCWs reported an IgG seropositivity duration of 415 days and 213 days for symptomatic and asymptomatic individuals, respectively; with obesity, age (over 55 years), and non-Caucasian ethnicity associated with longer seropositivity. ^38^ Finally, ethnicity is also a factor affecting seroprevalence with indigenous populations having the highest reported seroprevalence (ranging from 4.2 – 81.65%) and populations of African descent having 68% prevalence. ^35^^–^^37^ These differences might be explained by greater levels of migration (particularly in seeking work) and more menial work opportunities that don’t allow self-isolation, and a consequently greater risk of exposure to SARS-CoV-2. This is exemplified by a study done in Colombia where the global prevalence in the populations studied was 32.25%, while in Pacific coastal city of Guapi (where a high proportion of the population is of African descent with high rates of temporary migration), the IgG seroprevalence was 68%. ^10^

There are relatively few longitudinal data on mucosal antibody (IgA) response to SARS-CoV-2 from settings in low and middle-income countries. A systematic review of 38 original studies measuring specific IgA reported a seroprevalence of 86.5% among PCR positive SARS-CoV-2 patients and that the production of specific IgA was detectable during the first 10 days following infection, and remained detectable for prolonged periods (e.g. 75 days after symptom onset). ^39^ Finally, a study that analysed 100 communities in Wuhan, China showed that the presence of specific IgA antibodies decreased rapidly over time, as for IgM, especially those infected but who were asymptomatic. ^40^

Our study will evaluate differences in seroprevalence between cohorts with different occupational exposures (HCWs, highly mobile workers, and university staff and students) believed to have differing a priori risks of exposure to SARS-CoV-2. To our knowledge, there are no published longitudinal studies analysing cohorts with differing occupational risks for changes in systemic and mucosal antibodies to SARS-CoV-2. We hope to identify relevant factors related to changes in antibody levels including the effect of vaccination (including vaccine type and dose), occupation, and natural infections (and re-infections) with SARs-CoV-2.

### Study limitations

The study is subject to several potential limitations. Significant losses to follow-up can lead to selection bias and these are being minimized by close coordination of project activities with community leaders and institutional authorities, and regular contacts with study participants (at 3-monthly intervals). Recall bias is being minimized through these regular follow-ups to collect information on potential infections with SARS-CoV-2 and vaccination dates. Misclassification of data collection is being minimized by using a widely used standardized instrument (ISARIC WHO), and of antibody measurements by the use of validated detection assays blind to potential exposures. Because of high expected seroprevalence through vaccination, we are measuring antibody levels, thus maximizing the potential power of the study. Misclassification of exposure to SARS-CoV-2 is unavoidable and may be systematic because of limited access to PCR-based assays – underreporting of confirmed infections will likely be least in the hospital-based cohort. Similarly, the use of different vaccines of differing immunogenicity likely will influence maximal antibody levels: vaccine type and dose will be considered as a potential confounder or determinant in different longitudinal analyses. The receipt of multiple vaccine doses at regular intervals (up to 3 doses during 12 months or so of follow-up) may limit the ability to measure rates of decline in antibody levels.

## Conclusions

We are undertaking a prospective longitudinal cohort study to evaluate the effects of SARS-CoV-2 and vaccine exposures on longitudinal changes in systemic (plasma IgG) and mucosal (salivary IgA) antibodies to SARS-CoV-2. To our knowledge, this is one of very few such longitudinal studies being done in a low-middle income country setting and will contribute to our understanding of the impact of natural infectious exposures versus vaccination on population immunity, and the need for further booster vaccinations in different population groups.

## Data availability

No data are associated with this article.
