# Supplementary material for: The dynamics and determinants of specific systemic and mucosal antibody responses to SARS-CoV-2 in three adult cohorts in the Ecuadorian Andes: a study protocol
Source: F1000Res. 2024 Feb 23;11:1392. Originally published 2022 Nov 28. [Version 2] doi: 10.12688/f1000research.126577.2 (PMC10905138; doi:10.12688/f1000research.126577.2)
Supplement: Peer review history

# Reviewer report: Reviewer response for version 2

**DOI:** 10.5256/f1000research.162983.r250296

**Published:** 2024-02-26

**Authors:** Eleonora Nicolai

**Affiliations:** 1 Department of Experimental Medicine, University of Rome Tor Vergata, Rome, Italy

I appreciated your work highlighting the importance of the change in T lymphocytes and antibodies for different immune conditions. I found your work well written. I have only a few minor concerns:

- In the Contribution to the field paragraph, there are few typo errors:
- "In this work, we evaluated **that **the impact of preexisting immunity on the cellular immunity and humoral immunity in COVID-19 patients." Delete "that".
- "Further comparison found that the duration of hospitalization and the coronavirus negative conversion time in the mixed immunity subgroup were also shorter than those in the **to the **natural immunity subgroup and the acquired immunity subgroup." Delete "to the".
- In figure 3 are reported values within 4 weeks. It would be interested to extend the monitoring time.

Is the study design appropriate for the research question?

Yes

Is the rationale for, and objectives of, the study clearly described?

Yes

Are sufficient details of the methods provided to allow replication by others?

Yes

Are the datasets clearly presented in a useable and accessible format?

Yes

Reviewer Expertise:

NA

I confirm that I have read this submission and believe that I have an appropriate level of expertise to confirm that it is of an acceptable scientific standard.

# Reviewer report: Reviewer response for version 1

**DOI:** 10.5256/f1000research.139005.r216384

**Published:** 2023-11-15

**Authors:** Ria Lassaunière

**Affiliations:** 1 Department of Virus & Microbiological Special Diagnostics, Statens Serum Institute, Copenhagen, Denmark

The well-written protocol addresses relevant questions regarding antibody dynamics, factors associated with seroconversion, longitudinal changes in IgG and IgA levels, and the effect of vaccination on waning antibody levels. A strength of the study is the longitudinal design, inclusion of participants residing in different regions of Ecuador and of various occupations, and assessment of mucosal antibody responses. The authors address the limitations in the study including recall bias using interviews to collect data on a 3-monthly basis; however, it may be difficult to overcome this bias for exposures before enrolment. Furthermore, studying anti-spike antibodies only will not be sufficient to distinguish between infection and vaccination.

 Specific comments:

 Specific objective 3 – in addition to the factors listed, vaccine modality is also an important factor to consider. The authors list different vaccines administered to the Ecuadorian population including a whole inactivated vaccine (CoronaVac), mRNA vaccine (Pfizer-BioNTech), and adenoviral-vectored vaccines (AstraZeneca and Cansino), that could be considered.

 As the authors mention, it will not be possible to differentiate between infection-induced and vaccination-induced antibodies using anti-spike antibodies. With the exception of whole inactivated vaccines, approved COVID-19 vaccines do not include the nucleocapsid protein, a viral protein that induces antibody responses in those who become infected. Did the authors consider using an anti-nucleocapsid ELISA in addition to distinguish vaccine-induced responses from infection-induced responses?

 The risk of SARS-CoV-2 infection for farmers/merchants and university students/staff may be quite difficult to segregate considering the multitude of other factors contributing to transmission/acquisition of SARS-CoV-2, beyond the possibility to self-isolate. For example, farmers and merchants likely spend more time outdoors, with short interactions with other people in well-ventilated areas, unlike students who are much more social and aggregate in environments with a large number of people for longer periods of time, likely poorly ventilated environments. Since the authors will consider cohort membership for identifying risk factors for baseline seroprevalence, is it necessary to categorize the cohorts according to exposure risk?

 Do the authors anticipate a confounding effect of the antigen used in the ELISA after individuals may have been infected with Omicron that contains >12 amino acid changes in the RBD?

 It is unclear if this study protocol is written for a prospective study with planned recruitment and sampling, a retrospective study with planned antibody analyses on samples already collected, or an ongoing study. At the time of writing, were the cohorts being recruited (page 6), or were recruited and sample collection done and subjects contacted (page 8)?

Is the study design appropriate for the research question?

Yes

Is the rationale for, and objectives of, the study clearly described?

Yes

Are sufficient details of the methods provided to allow replication by others?

Partly

Are the datasets clearly presented in a useable and accessible format?

Not applicable

Reviewer Expertise:

Infectious diseases, Virology, Immunology

I confirm that I have read this submission and believe that I have an appropriate level of expertise to confirm that it is of an acceptable scientific standard, however I have significant reservations, as outlined above.

# Reviewer report: Reviewer response for version 1

**DOI:** 10.5256/f1000research.139005.r191772

**Published:** 2023-08-01

**Authors:** Chun-Yang Lin

**Affiliations:** 1 The University of Tennessee Health Science Center, Memphis, TN, USA

In the study protocol, Leon-Rojas et al. proposed a longitudinal cohort study to follow 1000 adults with varying SARS-CoV-2 exposure and to monitor the change in antibody levels against SARS-CoV-2 spike. Participant demographic information and baseline samples are collected at month 0. The clinical samples and relative information will be collected over the following 3, 6 and 12 months or when the participant receive the booster dose after 30-45 days. The antibody level in the samples is measured by ELISA and calibrated to NIBSC positive samples. With the study they want to understand 1) whether there are risk factors related to the level of SARS-CoV-2 exposure. 2) the change in SARS-CoV-2 spike antibody levels over a period of at least 12 months. 3) whether there are determinants that can affect antibody levels; 4) whether vaccination or other determinants affect antibody regression. 5) whether the relevant determinant also influences the SARS-CoV-2 IgA level in saliva. The results are analyzed by generalized estimating equations to construct a multivariable model for determinant identification.

 Major points:

- The introduction provides a comprehensive overview of the seroprevalence studies that have been conducted in Latin America. However, the rationale for the study is not explicitly stated until the discussion section. It would be helpful to emphasize the unique aspects of this study, such as the fact that it is a longitudinal study being conducted in a low-middle income country, in the introduction. This would help to make the study more relevant to the target audience.
- The authors propose to use SARS-CoV-2 RBD ELISA to determine the determinants of antibody levels. However, it is important to note that the antibody levels to SARS-CoV-2 RBD can be influenced by both natural infection and vaccination. If the authors do not closely monitor the participants, such as by conducting routine nasal swabs for PCR detection, the effect of vaccination or asymptomatic infection on the change in antibody levels could be confounded. It is therefore recommended that the authors include the detection of antibody to viral internal proteins, such as N, ORF8, or M proteins in the ELISA (1-2), in order to distinguish between the effects of infection and vaccination.
- The authors describe the methods of ELISA measurement using two reference papers. However, it would be helpful to provide more detail about the procedure, such as whether the serum will be serially diluted for the measurement and what TMB substrate will be used. Additionally, the authors should consider the huge different antibody levels that could be observed between vaccination and natural infection, which might out of ELISA detection range. If all samples are diluted to the same extent, the differences between the antibody levels in the vaccinated individuals may be limited because of saturating antibody binding.
- The authors mention that demographic and related information will be collected using WHO CRF Questionnaires. However, they do not specify which determinants they plan to include in the analysis. It would be helpful to provide more detail about the specific determinants that the authors plan to investigate, as this information would be relevant to the target audience.
  ​​

Overall, the study protocol is well-designed and has the potential to provide valuable insights into the determinants of antibody levels against SARS-CoV-2 in a low-middle income country. The suggestions that I have made are intended to improve the clarity and relevance of the study.

Is the study design appropriate for the research question?

Yes

Is the rationale for, and objectives of, the study clearly described?

Partly

Are sufficient details of the methods provided to allow replication by others?

Partly

Are the datasets clearly presented in a useable and accessible format?

Not applicable

Reviewer Expertise:

Mucosal immunology, virology, host-pathogen interactions, B cell, transcriptome analysis, influenza virus, SARS-CoV-2

I confirm that I have read this submission and believe that I have an appropriate level of expertise to confirm that it is of an acceptable scientific standard, however I have significant reservations, as outlined above.

## References

[1] : Antibody Response to SARS-CoV-2 Membrane Protein in Patients of the Acute and Convalescent Phase of COVID-19. *Front Immunol* .2021;12: 10.3389/fimmu.2021.679841 679841 10.3389/fimmu.2021.679841 34421894 PMC8371319

[2] : Accurate Diagnosis of COVID-19 by a Novel Immunogenic Secreted SARS-CoV-2 orf8 Protein. *mBio* .2020;11(5) : 10.1128/mBio.02431-20 10.1128/mBio.02431-20 33082264 PMC7587431
